# Real world experience with [^68^Ga]PentixaFor PET/CT in Primary Aldosteronism using newly developed harmonized diagnostic criteria

**DOI:** 10.3389/fendo.2026.1787233

**Published:** 2026-03-03

**Authors:** Linus Hesse, Anna Herden, James MacFarlane, Daniel Gillett, Tina Kienitz, Sophie Howarth, Loreen Richter, Dominik Soll, Dominik Spira, Frederike Butz, Martina T. Mogl, Federico Collettini, Christian J. Strasburger, Lukas Maurer, Knut Mai, Mark Gurnell, Christian Furth, Linus Haberbosch

**Affiliations:** 1 Department of Endocrinology and Metabolism, Charité – Universitätsmedizin Berlin, corporate member of Freie Universität Berlin, Humboldt-Universität zu Berlin, and Berlin Institute of Health, European Reference Network on Rare Endocrine Diseases (ENDO-ERN), Berlin, Germany; 2 Cambridge Endocrine Molecular Imaging Group, Institute of Metabolic Science, University of Cambridge and National Institute for Health Research Cambridge Biomedical Research Centre, Addenbrooke’s Hospital, Cambridge, United Kingdom; 3 Pituitary Tumor Center of Excellence at Charité – Universitätsmedizin Berlin, corporate member of Freie Universität Berlin, Humboldt-Universität zu Berlin, and Berlin Institute of Health, Berlin, Germany; 4 Department of Nuclear Medicine, Charité – Universitätsmedizin Berlin, corporate member of Freie Universität Berlin, Humboldt-Universität zu Berlin, and Berlin Institute of Health, Berlin, Germany; 5 Endocrinology in Charlottenburg, Berlin, Germany; 6 Department of Surgery, Charité – Universitätsmedizin Berlin, corporate member of Freie Universität Berlin, Humboldt-Universität zu Berlin, and Berlin Institute of Health, Berlin, Germany; 7 Department of Radiology, Charité–Universitätsmedizin Berlin, corporate member of Freie Universität Berlin, Humboldt-Universität zu Berlin, and Berlin Institute of Health, Berlin, Germany; 8 Department of Human Nutrition, German Institute of Human Nutrition Potsdam-Rehbrücke, Nuthetal, Germany

**Keywords:** [68Ga]PentixaFor, adrenal molecular imaging, adrenal vein sampling, positron emission tomography, primary aldosteronism

## Abstract

**Background:**

Positron emission tomography (PET) of the adrenal glands emerged as a non-invasive alternative to adrenal vein sampling (AVS) for treatment selection in primary aldosteronism (PA). The C-X-C-chemokine-receptor type 4 targeting tracer [^68^Ga]PentixaFor showed potential in visualizing aldosterone-producing adenomas. However, standardized diagnostic criteria and experience in European cohorts remain scarce. Here, we report harmonized PET/CT interpretation criteria, assess their impact on diagnostic agreement, and present a large European tertiary center’s experience with [^68^Ga]PentixaFor PET/CT in PA.

**Methods:**

Between 11/2023 and 01/2025, 35 consecutive PA patients underwent [^68^Ga]PentixaFor PET/CT; 22 also underwent AVS. PET/CT scans were interpreted in three independent, blinded reads, in which inter-reader agreement was assessed: (i) the routine clinical read by the local team, (ii) an independent review by external molecular imaging experts, (iii) a local re-evaluation using harmonized criteria adapted from experience with [^11^C]Metomidate PET. Surgical outcomes were analyzed according to Primary Aldosteronism Surgery Outcome (PASO) criteria.

**Results:**

Agreement between local and external readings was 80% (28/35), increasing to 94% (33/35) after applying harmonized PET/CT criteria. Based on local readings, 18/35 scans (51%) were interpreted as unilateral. In contrast, both the external review and the local team’s re-evaluation classified 11/35 (31%) as high probability, and 10/35 (29%) as intermediate probability of unilateral PA. According to local AVS criteria, 10/14 interpretable AVS indicated unilateral disease, with 8/10 concordant on PET. Combining successfully and partially cannulated AVS with PET findings, high confidence to diagnose unilateral disease increased to 12/22 (55%) patients. Twelve patients underwent adrenalectomy with PASO outcomes assessed ≥ 6 months after surgery, identified by AVS, PET or both (n=3, n=8 and n=1, respectively). Complete biochemical remission occurred in 2/3 operated patients based on AVS, 6/8 operated patients informed by PET, and 1/1 guided by both.

**Conclusion:**

[^68^Ga]PentixaFor PET continues to show promise for non-invasive PA subtyping. Harmonized interpretation criteria substantially improved inter-reader agreement. When combined with (partial) AVS, PET increases diagnostic confidence and may expand access to definitive treatment. Further studies are warranted to validate the proposed PET interpretation criteria and better define the subset of patients in whom [^68^Ga]PentixaFor PET/CT alone might suffice for PA subtyping.

## Introduction

Primary Aldosteronism (PA) is the most common potentially curable cause of hypertension, with a worldwide prevalence estimated between 5-14% among all patients with hypertension (1, 2). Although extensive evidence has demonstrated a higher risk of end-organ damage in PA compared with essential hypertension, it remains vastly underdiagnosed, with approximately 98% of all expected cases not diagnosed and treated (1–3).

Adrenal vein sampling (AVS) is considered the current gold standard diagnostic investigation to distinguish unilateral PA, which is potentially curable with surgery, from bilateral disease which currently requires lifelong medical therapy. However, AVS is an invasive and technically challenging procedure, and may not always yield conclusive results (1, 4). Additionally, the requirement to discontinue potentially interfering medication can deter patients from completing the diagnostic pathway towards potential cure (1, 2).

Molecular imaging of the adrenal glands offers a promising, non-invasive alternative to facilitate disease subtyping and optimize treatment selection for PA (5, 6). In particular, [^11^C]Metomidate (MTO) PET has been shown to be non-inferior to AVS for PA subtyping in the prospective, within-patient, MATCH clinical trial (5, 7); however, carbon-11’s short half-life of 20 min limits its availability to centers with an on-site cyclotron. Accordingly, other radioligands with a longer half-life are attracting interest, including an 18-fluorinated alternative to Metomidate - para-chloro-2-[^18^F]fluoroethyletomidate ([^18^F]CETO) and the unrelated C-X-C chemokine receptor type 4 (CXCR4) targeting tracer [^68^Ga]PentixaFor. The latter can be produced with a gallium generator and therefore does not necessarily require access to a cyclotron (8, 9). Two recently published systematic reviews have synthesized substantial evidence for its use in PA cohorts in China (10, 11). However, heterogeneous acquisition and reporting protocols have produced wide variation in published diagnostic accuracies, and the “optimal” quantitative PET metrics proposed to guide adrenalectomy have not been reproducible across centers. As recently proposed for MTO (MacFarlane et al., manuscript submitted), harmonized diagnostic criteria would likely benefit decision making, particularly with respect to confidence when recommending unilateral adrenalectomy.

Here, we report harmonized PET/CT interpretation criteria and our real-world experience with [^68^Ga]PentixaFor PET/CT for PA subtyping. We assess the impact of the reported PET/CT interpretation criteria on inter-reader agreement and evaluate how integrating [^68^Ga]PentixaFor PET with complete or partial AVS findings influences current clinical practice at our center (MacFarlane et al., manuscript submitted).

## Methods

### Patients

Between November 2023 and January 2025, 35 consecutive patients diagnosed with PA underwent [^68^Ga]PentixaFor imaging at our center. Written informed consent for all interventions and diagnostic procedures, including [^68^Ga]PentixaFor PET/CT was obtained from all patients. This retrospective study was approved by the institutional research committee (Ethikkommission der Charité - Universitätsmedizin Berlin - EA1/032/25) and the ethics committee of the Ärztekammer Berlin (Eth-S-R/14).

### Diagnosis

All patients had a documented elevated aldosterone-renin-ratio (ARR) above the local reference of >20 [ng*L^−1^/ng*L^−1^] with interfering medication known to cause false-positive results appropriately paused prior to testing. In 28 (80%) of the patients, the PA diagnosis was confirmed via saline infusion tests. In the remaining 7 patients (20%), undetectable renin concentrations, spontaneous hypokalemia and plasma aldosterone concentration (PAC) >200pg/ml established the diagnosis in line with the 2016 Endocrine Society criteria (12).

### Clinical care and outcomes

Treatment recommendations by the local multidisciplinary team (nuclear medicine physicians, radiologists and endocrinologists) were based on a combination of PET/CT and AVS results (available in 22 patients). Postoperative outcomes were analyzed at least 6 months after surgery in accordance with the Primary Aldosteronism Surgery Outcome (PASO) criteria (13). All patient care followed local and international clinical guidelines.

### AVS

A subgroup of 22 patients also underwent AVS, which was performed without adrenocorticotropic hormone (ACTH) stimulation. Cannulation was considered successful for a selectivity index > 2 (compared to a simultaneously drawn sample in the low inferior vena cava [IVC]). In routine clinical practice, bilateral successful cannulation resulting in a lateralization index (LI) >2 was interpreted as unilateral disease. If only a single adrenal vein was cannulated, a contralateral suppression index <1 was also interpreted as indicative of unilateral disease. Unilateral cannulation without contralateral suppression was not used to exclude any subtype.

**Figure 1:**
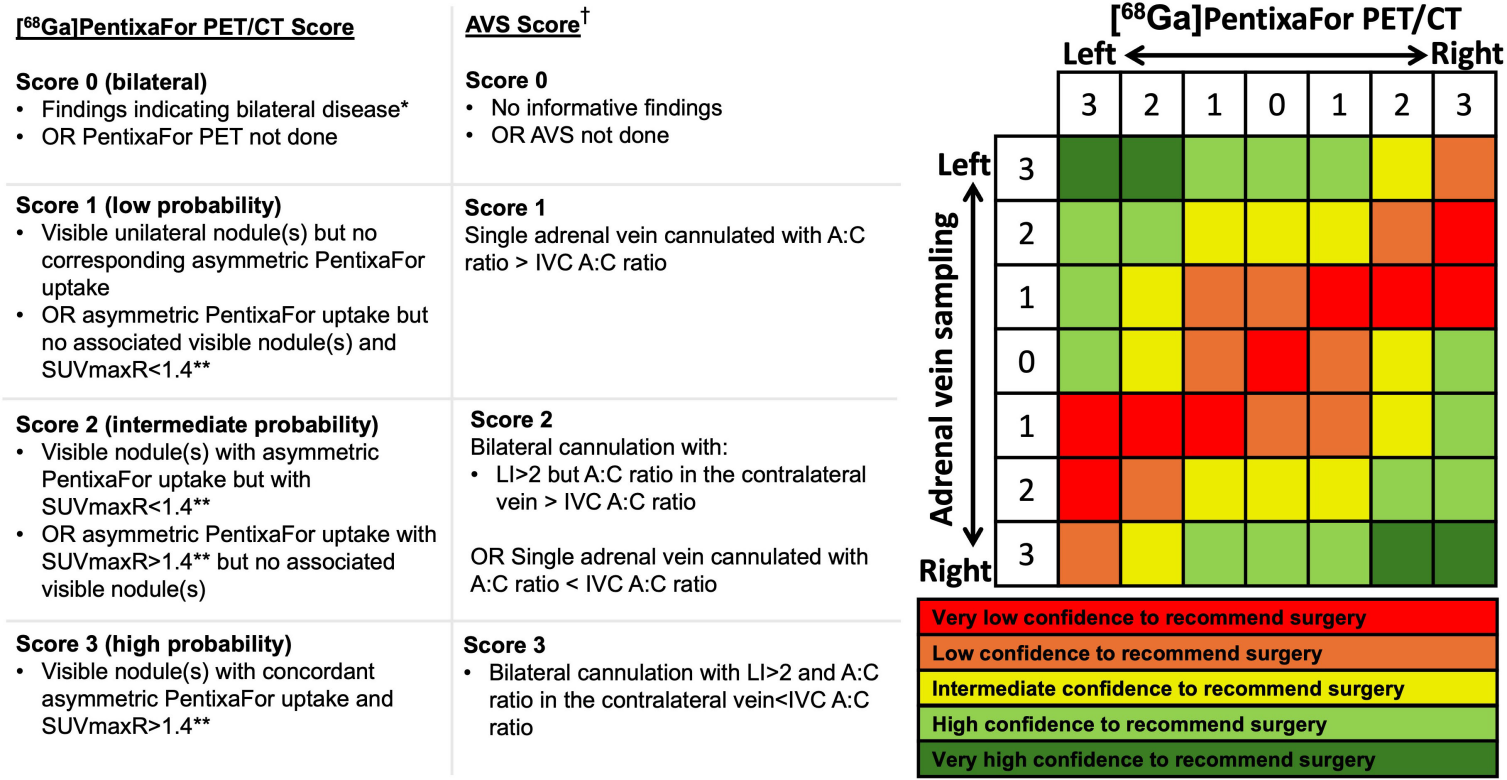
[^68^Ga]PentixaFor PET/CT and AVS scoring criteria and the decision matrix representing differing confidence levels to recommend surgery. The matrix integrates PET and AVS findings to represent the underlying confidence of supporting adrenalectomy. PET scores are translated horizontally and AVS scores vertically. Each point assigned for unilateral PA from the left adrenal gland shifts the position leftwards (for the PET score) or upwards (for the AVS score), whereas each point assigned to the right adrenal shifts the position rightwards (PET) or downwards (AVS). The final cell therefore represents the combined underlying confidence to recommend unilateral surgery. ^†^ based on non-ACTH-stimulated AVS at our centre; given the heterogeneity in AVS protocols and cut-offs, AVS results should be interpreted using the centre’s predefined criteria (14, 15).* if an investigation is highly indicative of bilateral disease one can consider to proceed with medical treatment. Current Endocrine Society guidelines consider AVS the most accurate currently available option for lateralization (14). **compared to the contralateral adrenal. Adapted from MacFarlane et al., manuscript submitted. SUVmaxR, SUVmax ratio (dominant/non-dominant); A:C ratio, aldosterone-to-cortisol ratio; IVC, inferior vena cava; LI, lateralization index.

The harmonized scoring matrix for combining PET/CT and AVS findings (**Figure 1**) scored AVS results in a standardized fashion: Score 0, AVS not done or no informative findings; score 1, single adrenal vein cannulated with aldosterone-to-cortisol ratio (A:C ratio) > IVC A:C ratio; score 2, bilateral cannulation with: LI > 2 but A:C ratio in the contralateral vein > IVC A:C ratio or single adrenal vein cannulated with A:C ratio < IVC A:C ratio; score 3, bilateral cannulation with LI > 2 and A:C ratio in the contralateral vein < IVC A:C ratio.

### [^68^Ga]PentixaFor imaging and image analysis

PET/CT imaging was performed using a Gemini TF TOF 16 (Gemini TF 16; Philips, Amsterdam, The Netherlands) with Time-of-Flight (TOF) capability (Philips Astonish TF technology). Each patient received approximately 2.5 MBq/kg body weight of [^68^Ga]PentixaFor (mean activity, 184 MBq; range 150–205 MBq). All PET-derived quantitative analyses were based on whole-body acquisitions, at a mean of 76 minutes post-injection (range 52-117) with 2 minutes per bed position, which were performed consistently in all patients.

PET images were reconstructed using iterative reconstruction with TOF analysis (BLOB-OS-TF; iterations, 3; subsets, 33). The projection data was reconstructed with 4 mm slice thickness (matrix size: 144 x 144; voxel size, 4x4x4 mm). Attenuation correction CT images were reconstructed with a slice thickness of 5 millimeters (convolution kernel, B08). All scans were assessed both visually and semi-quantitatively (by calculation of maximum standardized uptake values [SUVmax]) by two local nuclear medicine physicians, who recorded the most likely PA subtype derived from PET/CT in the electronic report form. If not previously available, contrast-enhanced CT of the upper abdomen was performed on the PET/CT scanner.

### Retrospective reappraisal of PET and harmonized PET interpretation criteria

Subsequently, all PET/CT scans were reread by an external expert center (Cambridge, UK) for molecular imaging in PA. Blinded to AVS results, initial local reports and postoperative outcomes, each scan was classified as low, intermediate or high probability of unilateral disease; based on a [^68^Ga]PentixaFor-adapted version of the recently proposed scoring matrix by MacFarlane et al., that was originally developed and validated for MTO PET/CT (MacFarlane et al., manuscript submitted). The [^68^Ga]PentixaFor-adapted harmonized PET/CT lateralization criteria (**Figure 1**) were as follows: Bilateral disease (score 0): symmetric [^68^Ga]PentixaFor uptake indicating clear symmetric bilateral disease; low probability for unilateral disease (score 1): visible unilateral nodule(s) but no corresponding asymmetric [^68^Ga]PentixaFor uptake or asymmetric [^68^Ga]PentixaFor uptake but no associated visible nodule(s) and SUVmax ratio (SUVmaxR)<1.4; intermediate probability (score 2): visible nodule(s) with asymmetric [^68^Ga]PentixaFor uptake but with SUVmaxR<1.4 or asymmetric [^68^Ga]PentixaFor uptake with SUVmaxR>1.4 but no associated visible nodule(s); high probability (score 3): visible nodule(s) with concordant asymmetric [^68^Ga]PentixaFor uptake and SUVmaxR>1.4.

The adaption of the scoring system was based on the currently existing [^68^Ga]PentixaFor publications (systematically reviewed in **Supplementary Table 2**, **Supplementary Figures 1**, **2**) and our initial experience with [^68^Ga]PentixaFor PET as well as the local cut-offs for ACTH unstimulated AVS. After this external evaluation, the local team performed a re-evaluation using the same harmonized criteria, blinded to external reports.

Concordance between local and external reports was assessed both before and after the local team applied the harmonized criteria. First, reports were defined as concordant if (i) the local team documented the suspicion of unilateral disease, and the external center indicated either high or intermediate probability of unilateral disease on the same side or (ii) both local and external centers identified bilateral disease or low probability of unilateral disease. For the local re-evaluation of PET/CT scans using the harmonized criteria, a PET/CT score difference of >1 was rated as non-concordant to the external center evaluation.

## Results

### Cohort characteristics

The mean patient age at the time of scan was 45 years (range 26-75); 49% (n=17) of the patients were women. Adrenal abnormalities were observed on CT or MRI in 28 (80%) patients. The mean lesion size was 12.2 mm (range 4-27) across 21 adrenal glands, and an additional 17 adrenal glands were reported as bulky. Baseline characteristics are summarized in **Table 1**.

**Table 1:** Patient characteristics.

Characteristics	Entire cohort	
	*n*	Value
Age (yr)	35	45 ± 13
Female	17	49%
BMI (kg/m²)	35	27.8 ± 4.9
Systolic Blood Pressure (mmHg)	35	150 (136, 160)
Diastolic Blood Pressure(mmHg)	35	90 (85, 100)
Years since Hypertension was first detected	35	6 (1, 14)
Biochemistry		
Serum Potassium (mmol/L)	35	3.78 ± 0.42
History of spontaneous hypokalemia	19	54%
PAC (ng*L^−1^)	35	179 (151, 243)
PRC (ng*L^−1^)	35	2.6 (<1.1, 4.1)
ARR (ng*L^−1^/ng*L^−1^)	35	97 (52, 198)
PAC post saline infusion test	28	176 (123, 224)
Anatomical imaging		
Bilateral normal adrenals	7 (patients)	20%
Bilateral abnormalities	10 (patients)	29%
Unilateral abnormalities	18	51%
Lesion size (mm)	21 (lesions)	11 (8, 15)
Left sided lesions	13	62% of 21 lesions
Right sided lesions	8	38% of 21 lesions
Bulky adrenal glands	17 (from 13 patients)	24% of 70 glands

Data is presented as percent, mean ± SD or median (Quartile 1, Quartile 3).

PAC, Plasma Aldosterone Concentration; PRC, Plasma Renin Concentration; ARR, Aldosterone-Renin-Ratio.

### [^68^Ga]PentixaFor PET and AVS results

Based on the initial local reads (i), 18 patients (51%) were interpreted as having unilateral disease. SUVmax ratios in these 18 patients were significantly higher compared to those with bilateral disease (p = 0.0037). The external review (ii) rated 11 of 35 (31%) as having a high probability and 10 of 35 (29%) as having an intermediate probability of unilateral PA. Five of these 21 scans had initially been classified as bilateral in the local read (i), whereas two cases initially rated as unilateral in (i) received a PET/CT score of 1 (low probability) in the external evaluation (ii). Consequently, concordance between the initial local reports (i) and the external expert readings (ii) was observed in 28 of 35 cases (80%).

Following the second local review (iii) using the newly developed harmonized criteria (and blinded to the external center’s classifications), the concordance increased to 33/35 (94%) cases. The McNemar test comparing agreement before versus after harmonized criteria (non-concordant pairs: 0 worsened, 5 improved) yielded p=0.0625. In the remaining two cases, only one center assigned intermediate probability of unilateral disease; consensus was reached after discussion.

The smallest lesion deemed unilateral by PET was 7 mm in diameter on anatomical imaging. Further details are presented in **Table 2**.

**Table 2:** Characteristics between PA subtypes in PET as initially reported by the local team.

Characteristics	Unilateral in PET	Bilateral in PET	*P*-Value
Number of subjects	18 (51%)	17 (49%)	
Age (yr)	46 ± 15	45 ± 12	.698
Female	9 (50%)	9 (53%)	.867
BMI (kg/m²)	27.8 ± 4.9	28.4 ± 5.1	.723
Systolic Blood Pressure (mmHg)	150 (140, 156)	150 (135, 160)	.655
Diastolic Blood Pressure(mmHg)	98 (89, 100)	90 (80, 99)	.152
Biochemistry			
Serum Potassium (mmol/L)	3.65 ± 0.36	3.93 ± 0.44	.054
History of spontaneous hypokalemia	13 (72%)	6 (35%)	.028
PAC (ng*L^−1^)	203 (151, 237)	166 (107, 265)	.363
PRC (ng*L^−1^)	2.75 (1.58, 3.85)	4.1(3.25, 4.2)	.103
ARR (ng*L^−1^/ng*L^−1^)	130 (67, 291)	92 (35, 160)	.151
Anatomical imaging			
Bilateral normal adrenals	1 (5%)	6 (35%)	.028
Bilateral abnormalities	3 (17%)	7 (41%)	.115
Unilateral abnormalities	14 (78%)	4 (24%)	<.001
Lesion size (mm)	13 (9.5, 16)	9.5 (8, 11.5)	.754
Bulky adrenal glands	9 (50%, from 7 patients)	8 (47%, from 6 patients)	.832
Left sided lesion	7 (39%)	6 (35%)	.832
Right sided lesion	4 (22%)	4 (23%)	.929
Molecular Imaging			
SUVmax Ratio	1.37 (1.21, 1.8)	1.09 (1.05, 1.17)	.004

Data is presented as percent, mean ± SD or median (Quartile 1, Quartile 3).

*P-*values are derived from two-tailed Student’s t-tests.

PAC, Plasma Aldosterone Concentration; PRC, Plasma Renin Concentration; ARR, Aldosterone-Renin-Ratio.

**Figure 2:**
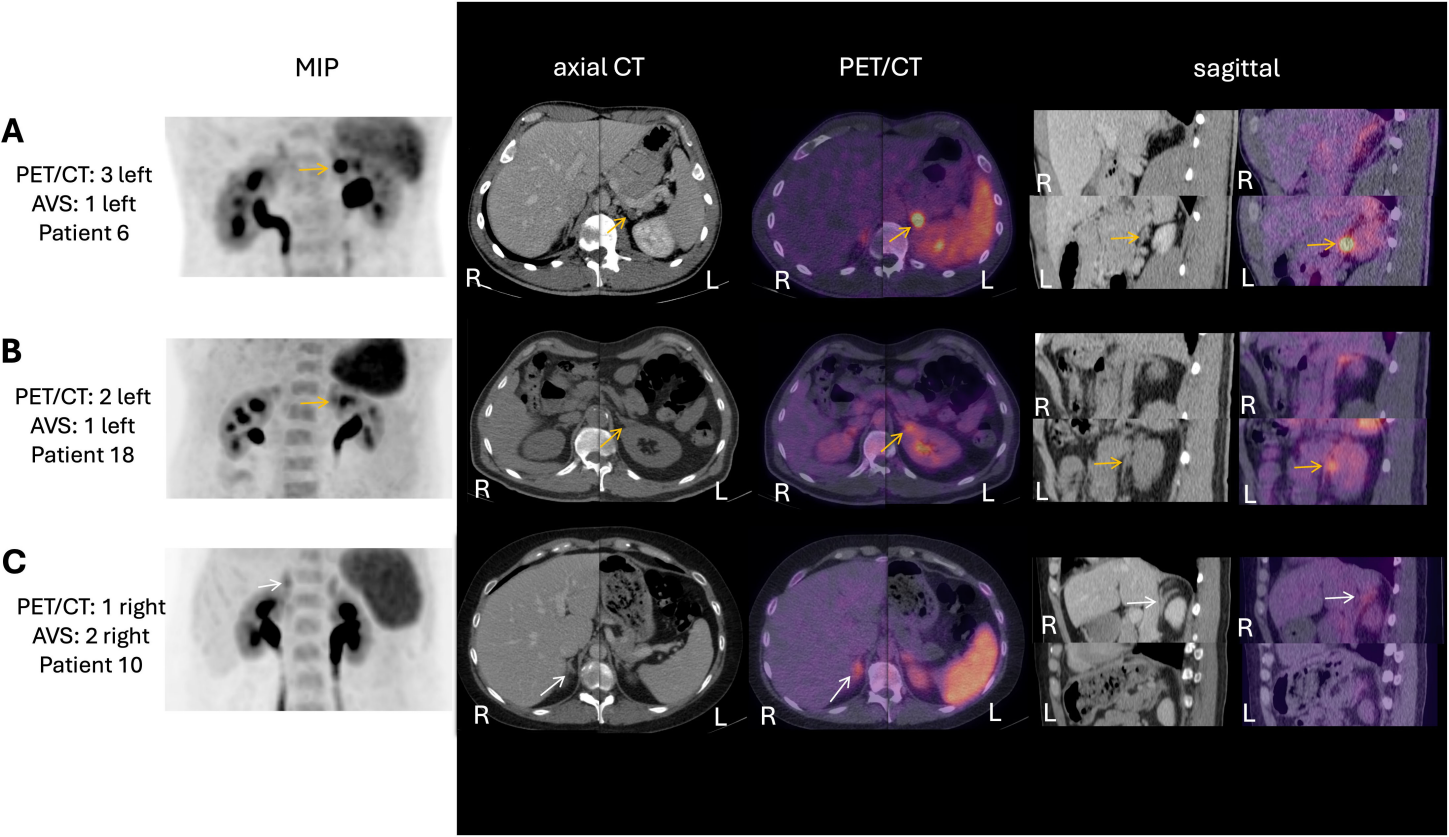
PET/CT images illustrating each PET scoring group. Multiplanar [^68^Ga]PentixaFor PET/CT images in three patients with their respective PET/CT and AVS scores. Yellow arrows indicate morphologically suspicious adrenal lesions with corresponding radiotracer uptake. White arrows indicate morphologically normal adrenal glands with asymmetric tracer uptake. **(A)** Failed cannulation of the right adrenal vein; left A:C ratio > IVC resulting in an AVS score of 1. PET/CT showed clear unilateral disease (score of 3) and guided partial left adrenalectomy of a 15 mm APA with complete biochemical and clinical success. **(B)** Failed right adrenal vein cannulation; left A:C ratio > IVC again results in an AVS score of 1. PET/CT - initially interpreted as unilateral PA - retrospectively yielded a PET/CT score of 2 to the same side. Consequently, if medical therapy was not well tolerated, concordant AVS and PET/CT findings would have supported counselling about a potential left-sided intervention, with the standardized matrix score transparently conveying the confidence level. The patient declined surgery and continued medical treatment. **(C)** AVS with lateralization index > 2 but no contralateral suppression results in an AVS score of 2. The PET/CT score of 1 supports the suspicion of asymmetric but bilateral disease enabling a discussion about potential treatment selection similar as in B). The patient declined surgery. MIP, maximum intensity projection; AVS, adrenal vein sampling; A:C ratio, aldosterone-to-cortisol ratio; IVC, inferior vena cava; R, right; L, left.

Of 22 patients who also underwent AVS, bilateral cannulation was successful in 12 (55%). In two additional patients (both with bilateral adrenal gland abnormalities on CT) cannulation of one adrenal vein demonstrated suppressed aldosterone secretion, yielding 14/22 (64%) interpretable AVS studies with 10/22 (46%) meeting the local criteria for unilateral disease. Among these 10 unilateral AVS cases, PET demonstrated high, intermediate or low probability of unilateral disease to the same side in n=3, n=2 (both reported as unilateral in the initial local report) and n=3 patients, respectively. PET findings in the two remaining cases suggested bilateral disease, and intermediate probability of unilateral disease on the contralateral side. Representative PET/CT images from each PET scoring group are shown in **Figure 2**.

In total, AVS and [^68^Ga]PentixaFor PET agreed on either asymmetric disease (lateralization towards the same side) or bilateral disease in 17/22 (77%) patients. However, in four patients, AVS indicated unilateral disease with at least intermediate probability, whereas [^68^Ga]PentixaFor PET suggested bilateral disease (pathways in the scoring system illustrated in **Supplementary Figure 5**). Notably, all adrenal glands in these discrepant cases were described as normal on anatomical imaging.

In the remaining case, [^68^Ga]PentixaFor PET and AVS indicated unilateral disease on opposing sides (with high probability for AVS and intermediate probability on PET).

Adverse events occurred in two AVS procedures (retroperitoneal hematoma, infection). No adverse events were associated with [^68^Ga]PentixaFor PET/CT.

### Combining data from [^68^Ga]PentixaFor PET with AVS

After AVS data was combined with [^68^Ga]PentixaFor PET findings, high confidence to diagnose unilateral disease increased from 10/22 (46%) patients to 12/22 (55%) patients who had both investigations. An additional six patients reached intermediate confidence to recommend surgery. The scoring system used for AVS as well as for [^68^Ga]PentixaFor PET is shown in **Figure 1**.

### Postoperative outcomes

By the time of analyses, twelve patients had proceeded to surgery with PASO outcomes assessed at least 6 months following adrenalectomy: three informed by AVS, one informed by PET and AVS, and eight informed by PET. According to PASO criteria at least 6 months after surgery, complete biochemical remission was achieved in 6/8 operated patients informed by PET, 2/3 operated patients based on AVS and 1/1 guided by both. Two patients showed partial biochemical remission; one of them is illustrated in detail, including why adrenalectomy was based on PET in the initial local report, in **Supplementary Figure 3** and **Supplementary Table 1**. One further PET guided patient with an intermediate probability of unilateral disease showed absent biochemical success.

**Figure 3:**
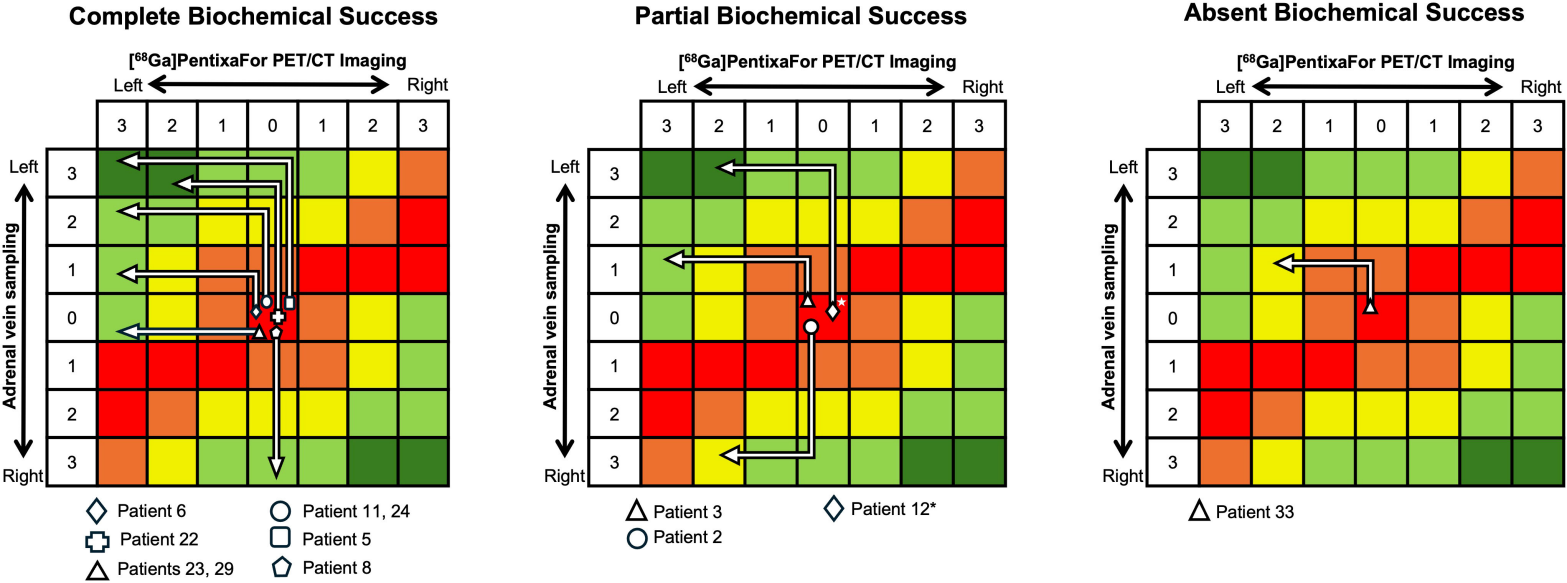
Pathways of all operated patients in the scoring matrix, grouped by their biochemical outcome. Each patient’s PET and AVS scores are mapped onto the matrix; the final cell represents the combined confidence level and the implied side of recommendation for unilateral adrenalectomy. Example: in patient 5, both modalities indicated a high probability of left-sided unilateral disease. An AVS score of 3 (left) shifts the position three cells upwards, and a PET score of 3 (left) shifts it three cells leftwards, placing the case in the upper-left corner of the matrix, indicating very high confidence to recommend left adrenalectomy. *In clinical practice, the AVS of patient 12 was interpreted locally as ambiguous; but retrospective application of the proposed AVS criteria yielded an AVS score of 3 (illustrated in detail in **Supplementary Table 1** and **Supplementary Figure 3**). The PET/CT was initially interpreted as unilateral left PA by the local team and guided left adrenalectomy. In the retrospective reappraisal with standardized PET/CT interpretation criteria, this case was assigned a PET score of 2 (intermediate probability) - a PET/CT interpretation more compatible with asymmetric bilateral PA, as reflected by partial biochemical and partial clinical remission.

The pathways of all operated patients in the scoring matrix, grouped by their biochemical outcome are presented in **Figure 3**. The clinical outcomes at least 6 months after surgery demonstrated complete clinical remission in 5/8 and partial remission in 3/8 patients operated based on PET; complete remission in one patient and partial remission in 2/3 patients operated based on AVS; and complete clinical remission in the patient guided by both modalities.

CYP11B2 immunohistochemistry was available in 10/12 resected specimens. Applying HISTALDO, the international consensus for histopathology in unilateral PA (16), identified five classical (n=4 solitary aldosterone producing adenomas [APA], and n=1 solitary aldosterone producing nodule [APN]), and five non-classical PA patterns (n=5 multiple aldosterone producing (micro-)nodules [MAPN]) (16). Outcomes differed markedly by histopathological subtype: 5/5 patients with classical pathology achieved complete biochemical success, and complete clinical success was observed in 4/5. In contrast, among non-classical cases, 2/5 achieved complete biochemical success, while 2/5 had partial and 1/5 absent biochemical success; clinically, complete success occurred in 1/5 and partial clinical success in 4/5.

SUVmax ratios tended to be higher in APAs than in MAPN (median 1.93, range [1.09-3.44] vs. 1.18 [1.17-1.68]). The maximum lesion size showed a low, non-significant association with SUVmax (Spearman’s ρ = 0.52, p = 0.12) and the SUVmax ratio (ρ = 0.44, p = 0.21).

High confidence to recommend surgery based on PET, more frequently yielded classical pathology: 4/5 classical cases had a PET score of 3, compared with 1/5 non-classical cases. The smallest PET-identified lesion that was resected was an 8-mm APN; additionally, two PET-informed surgeries revealed MAPNs up to 3 mm. In two adrenals resected based on AVS despite PET suggesting bilateral disease, histopathology demonstrated MAPN with lesions up to 1 mm in diameter.

## Discussion

In this study, we present our real-world experience with [^68^Ga]PentixaFor PET/CT for PA subtyping. Our findings demonstrate a high level of inter-reader agreement when using the adapted scoring system for classifying the likelihood of unilateral disease in [^68^Ga]PentixaFor PET. We further validate the clinical benefit of combining (partial) AVS results with [^68^Ga]PentixaFor PET. Our data highlights how PET can facilitate broader access to definitive surgical treatment, particularly in centers where access to conclusive AVS is limited.

As the majority of patients suffering from PA are currently not diagnosed and treated, there is a need for subtyping methods that can be readily implemented across multiple clinics treating patients with hypertension (1, 2). However, broader adoption of [^68^Ga]PentixaFor PET/CT will likely require a standardized interpretation system especially in centers with relatively low case numbers of both PET and AVS. Standardized reporting could improve clinicians’ confidence in making informed decisions to recommend interventions solely based on PET/CT, potentially allowing PET/CT to replace AVS as a cost-efficient subtyping modality for a large proportion of patients with PA (17). Expanding access to curative treatment might not only improve cardiovascular outcomes but also enhance quality of life compared to PA patients on medical treatment (18).

### PET interpretation

Since the first report of [^68^Ga]PentixaFor PET as a non-invasive method for visualizing APAs in 2018 (8), multiple studies have documented high diagnostic performance (the results of a systematic review are provided in **Supplementary Table 2**) (8, 9, 17, 19–38). However, considerable variation exists in the optimal cut-off values and their reported sensitivities and specificities. Proposed semiquantitative parameters include: (i) the SUVmax of the lesion itself, (ii) the lesion-to-liver SUVmax ratio (LLR), (iii) the lesion-to-ipsilateral adrenal gland SUVmean ratio (LAR), (iv) the lesion-to-contralateral adrenal gland SUVmean ratio (LCR), and (v) the lesion-to-contralateral adrenal gland SUVmax ratio, sometimes referred to as the PET lateralization index (LI). The four largest studies, each consisting of ≥100 PA patients who underwent [^68^Ga]PentixaFor PET do not report higher discriminatory value for semiquantitative parameters that use liver uptake for normalization compared to visual analysis or adrenal SUV based subtyping (9, 20, 21, 32). The currently largest study investigating 208 patients with PA demonstrated highest diagnostic performance for an SUVmax ratio cut-off of 1.5 and visual analysis [both with an area under the receiver operating characteristic curve (AUC) of 0.82 while the AUC for the LLR was 0.71], compared with the PA subtype based on AVS and postoperative outcomes, where available (20).

The large variability in reported optimal cut-off values for semiquantitative parameters - ranging from 4.71-11.18 for the single SUV detecting functional lesions, 2.17-6.7 for the LLR and 1.39-3.6 for the LCR (8, 9, 19–29, 32, 33, 37), demonstrates their dependence on different imaging protocols. In addition, a substantial influence from the specific PET system used must be expected including differences between analog and digital scanners, variations in spatial resolution and vendor-specific reconstruction algorithms. Such algorithms are often designed to produce a harmonized visual impression but may involve pixel smoothing or even enhancement of certain image features, for example through point-spread-function-based reconstructions. Therefore, adherence to harmonization protocols such as the European Association of Nuclear Medicine (EANM) EARL accreditation standards is important when applying semi-quantitative PET parameters (39).

The variations may also represent heterogeneity in measuring parameters such as the adrenal gland- or liver SUVmean across different centers (40). Optimal cut-offs for LAR show less variability, ranging from 1.6-1.95. However, only three studies reported this parameter (21, 22, 32), with uncertainty regarding the respective SUVmean calculation (22). Another study applied the term LAR to the ratio of lesional SUVmax to *contralateral* adrenal gland SUVmean (38) - a metric that other studies, including the present one, refer to as LCR.

High reliability of semiquantitative parameters is essential for broader application across centers with different PET scanners and reporting software. Therefore, we decided to adapt the SUVmax ratio from both adrenal glands for [^68^Ga]PentixaFor PET. This method has been proven highly reliable for other tracers in PA across multiple centers (5, 41, MacFarlane et al., manuscript submitted). Furthermore, a ratio based on two SUVmax would be less susceptible for center or reporter specific influences compared to SUVmean calculations in clinical routine (40). The optimal cut-off values reported across all studies are illustrated in **Supplementary Figure 1**.

In our own series, harmonized PET interpretation criteria increased inter-institutional concordance from 80% (28/35) to 94% (33/35). Although a McNemar test demonstrated a p-value of 0.0.625, the lack of significance might be explained by the limited cohort size and the small number of discordant reads. Therefore, the rise in agreement underscores how a standardized approach can enhance the reliability of [^68^Ga]PentixaFor PET, giving clinicians greater confidence to guide informed decisions for further diagnostic work-up and subsequent treatment selection.

### PET and AVS

Reported concordance rates in published data with AVS range from 50% to 90% (presented in **Supplementary Figure 2**), with most studies - consistent with our findings – indicating values of 60-75% (21–23, 25), even when AVS success rates reach up to 98% (23). The relatively low concordance between [^68^Ga]PentixaFor PET and AVS, coupled with similar surgical success rates, echoes earlier findings for MTO PET (7). This suggests that AVS and PET may have complementary rather than mutually exclusive roles in future PA subtyping, highlighting the need for a systematic approach of combining information from both investigations. In cases where both PET and AVS independently suggest low to intermediate probability for long-term postsurgical improvement, a likely diagnosis is bilateral disease with an asymmetric dominant side. However, interventions such as (partial) adrenalectomy, ablation or selective arterial embolization may still be considered beneficial, particularly for patients whose medical therapy poses challenges (42). Importantly, relying on only one modality could prevent some patients from an informed shared decision about further treatment selection of a lifelong disease (MacFarlane et al., manuscript submitted).

With the currently limited knowledge regarding the optimal selection criteria for each diagnostic modality, patients who would be candidates for adrenalectomy might require both PET and AVS to prevent misclassification as bilateral disease if only one modality is used (7). Prospective trials will need to incorporate both modalities in their participants when determining the true diagnostic accuracy of each approach or compare novel tracers.

### Clinical decision-making

In clinical practice, it is essential to identify which subgroups of patients would benefit most from PET (and thus may not require AVS). Our results are consistent with two trials involving 100 and 208 patients, respectively, that report higher diagnostic certainty with PET for lesions ≥10 mm (9, 20). Two studies demonstrated a significant correlation (Spearman’s ρ= 0.61, Pearson’s r=0.53) between SUVmax and lesion diameter in 43 and 66 patients with unilateral PA (22, 23). Two patients which were identified as unilateral only on AVS showed normal adrenal morphology on anatomical imaging, possibly reflecting the limited spatial resolution of [^68^Ga]-labeled tracers due to their higher energy and longer positron range. In contrast, [^11^C] or [^18^F] with a more favorable decay profile and lower positron energy offer superior spatial resolution and may improve the detection of small lesions (43).

Conversely, two studies reported better diagnostic performance of [^68^Ga]PentixaFor PET for lesions <10 mm than for ≥10 mm lesions (22, 32), hypothesizing that smaller nodules have higher CYP11B2 expression, which has been correlated with CXCR4 (44). Further studies comparing [^18^F]-labelled tracers that target CXCR4 or CYP11B2 in direct comparison with AVS and [^68^Ga]PentixaFor PET will resolve these discrepancies (45–47). In parallel, improvements of digital PET systems and their reconstruction parameters are likely to increase the detectability of sub-centimeter lesions.

### Limitations

This retrospective study reporting routinely collected, heterogeneous clinical data has several limitations that constrain generalizability. Only 12/35 (34%) underwent adrenalectomy with available PASO follow-up, and among the 22/35 (63%) patients who underwent AVS, bilateral cannulation was successful in only 55%. Accordingly, the proposed harmonized PET/CT interpretation criteria are not primarily derived from this small cohort but were adapted from prior experience with [^11^C]MTO PET and a systematic review of the available [^68^Ga]PentixaFor literature (provided in the supplementary materials). Applying these criteria to our cohort illustrates the potential use case - improving reporting, inter-reader agreement and potentially also clinical decision-making for an emerging tracer - rather than constituting definitive validation. An assessment of definitive clinical utility is needed before wider use of the proposed criteria and will require larger, preferably prospective, surgically validated cohorts with standardized follow-up and HISTALDO correlation.

Furthermore, our cohort, like most cohorts represented in the current [^68^Ga]PentixaFor literature, may not reflect the full spectrum of PA. Patients referred for PET and/or AVS are a selected subgroup, with a higher pre-test probability of unilateral disease and generally more overt PA phenotypes, because these investigations should only be pursued after rigorous counselling in patients who would consider adrenalectomy if unilateral disease was confirmed. The limited representation of specific PA subgroups (e.g., multifocal, diffuse disease, and milder phenotypes) may also restrict generalizability of the proposed criteria.

## Conclusion

The presented data is consistent with previous findings and reinforce the potential of [^68^Ga]PentixaFor PET/CT as a non-invasive, complementary modality to AVS for PA subtyping. Combining findings from (partial) AVS and PET can expand access to definitive cure. If validated, the proposed interpretation criteria may ultimately allow [^68^Ga]PentixaFor PET/CT to replace AVS in a subgroup of PA patients. Further studies are warranted to confirm and refine harmonized PET criteria and better identify patients in which [^68^Ga]PentixaFor PET/CT alone might suffice for PA subtyping.

## Data Availability

The raw data supporting the conclusions of this article will be made available by the authors, without undue reservation.
