# Supplementary material for: Real world experience with [68Ga]PentixaFor PET/CT in Primary Aldosteronism using newly developed harmonized diagnostic criteria
Source: Front Endocrinol (Lausanne). 2026 Mar 3;17:1787233. doi: 10.3389/fendo.2026.1787233 (PMC12992006; doi:10.3389/fendo.2026.1787233)
Supplement: Supplementary file 1 [file DataSheet1.docx]

**Supplementary Material**

**Literature Review**

PubMed and Web of Science were systematically searched on the 14^th^ of December 2025 with the following Search strategy: *(PentixaFor OR CXCR4) AND aldosteron*.*

All results were screened for relevance by title and abstract. Subsequently, the full text was studied for eligibility. Case reports with n ≤ 3, conference abstracts already represented by full papers and articles not written in English or German were excluded. Relevant variables were extracted and are summarized in Supplementary Table 2. The study selection process is illustrated in the PRISMA flow chart in Supplementary Figure 6.


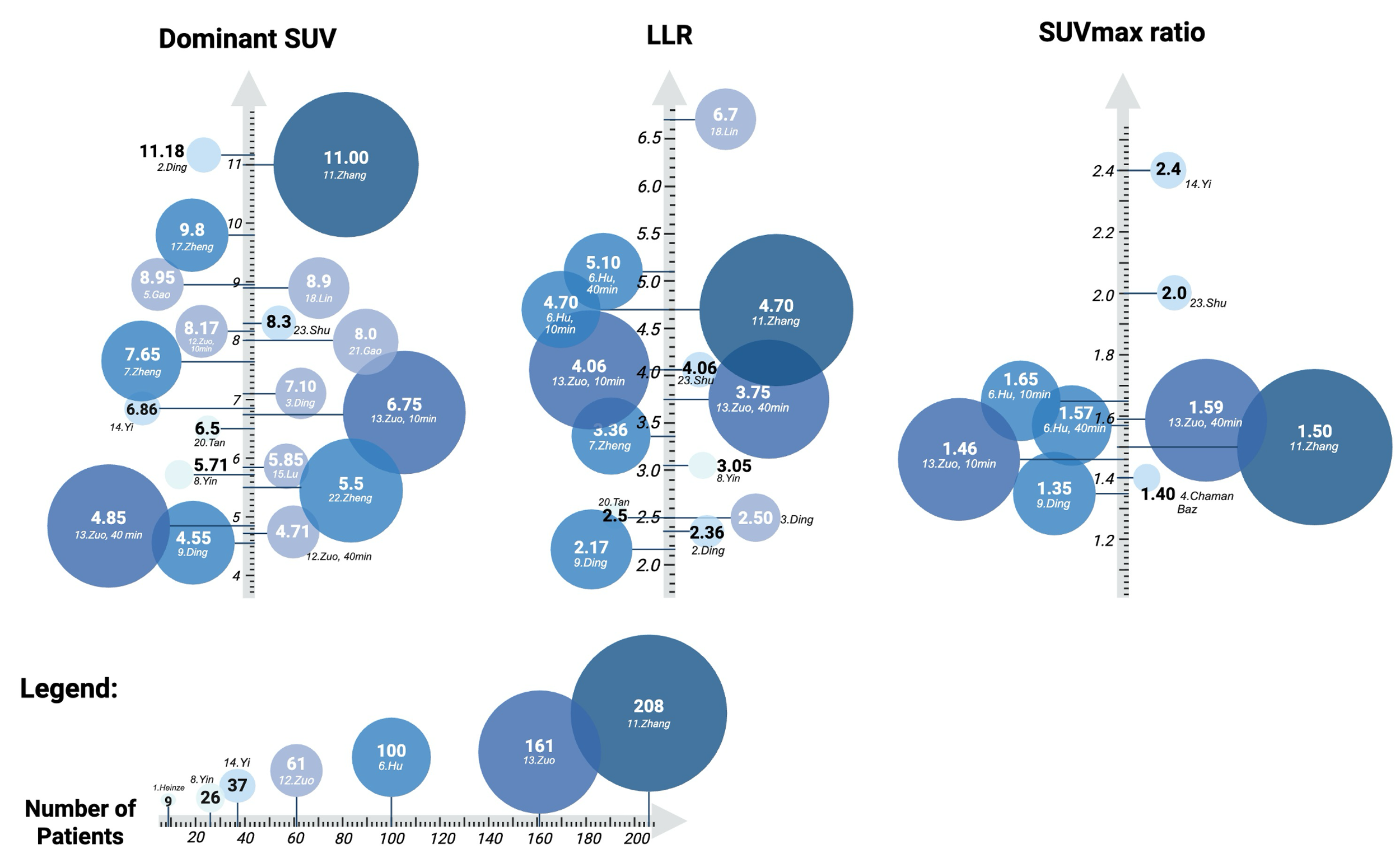
**Supplementary Figure 1.** Cut-off values reported as optimal across all studies investigating the respective semiquantitative parameter.

Illustration of the different cut-off values reported as optimal throughout all studies included in the systematic review. The circle size reflects the number of included patients in the respective study. Cut-off values reported as optimal are presented in the circles, adjacent to the first author and study number derived from Supplementary Table 2. SUV, Standardized uptake value; SUVmax, Maximum standardized uptake value; LLR, Lesion to liver ratio

**Supplementary Figure 2.** Reported AVS concordance across all studies.


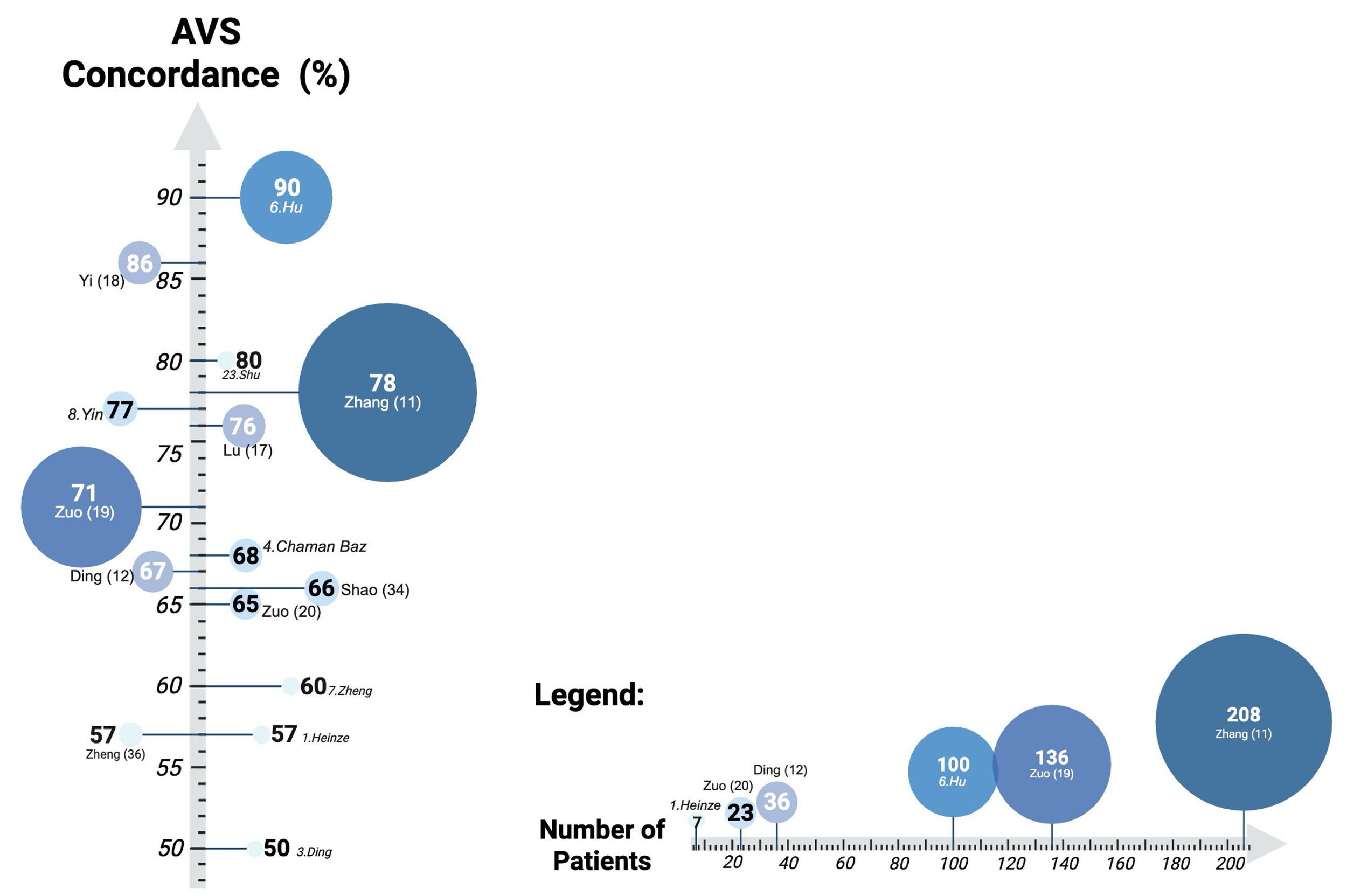


Illustration of the reported AVS concordance throughout all studies included in the systematic review. The circle size reflects the number of included patients in the respective study. The reported AVS concordance given in percentage is presented in the circle adjacent to the first author and study number derived from Supplementary Table 2. Circle sizes may vary compared to Supplementary Figure 1, as here, only the number of patients who also underwent AVS are represented.

**Supplementary Figure 3.** PET/CT of patient 12 with partial biochemical success.


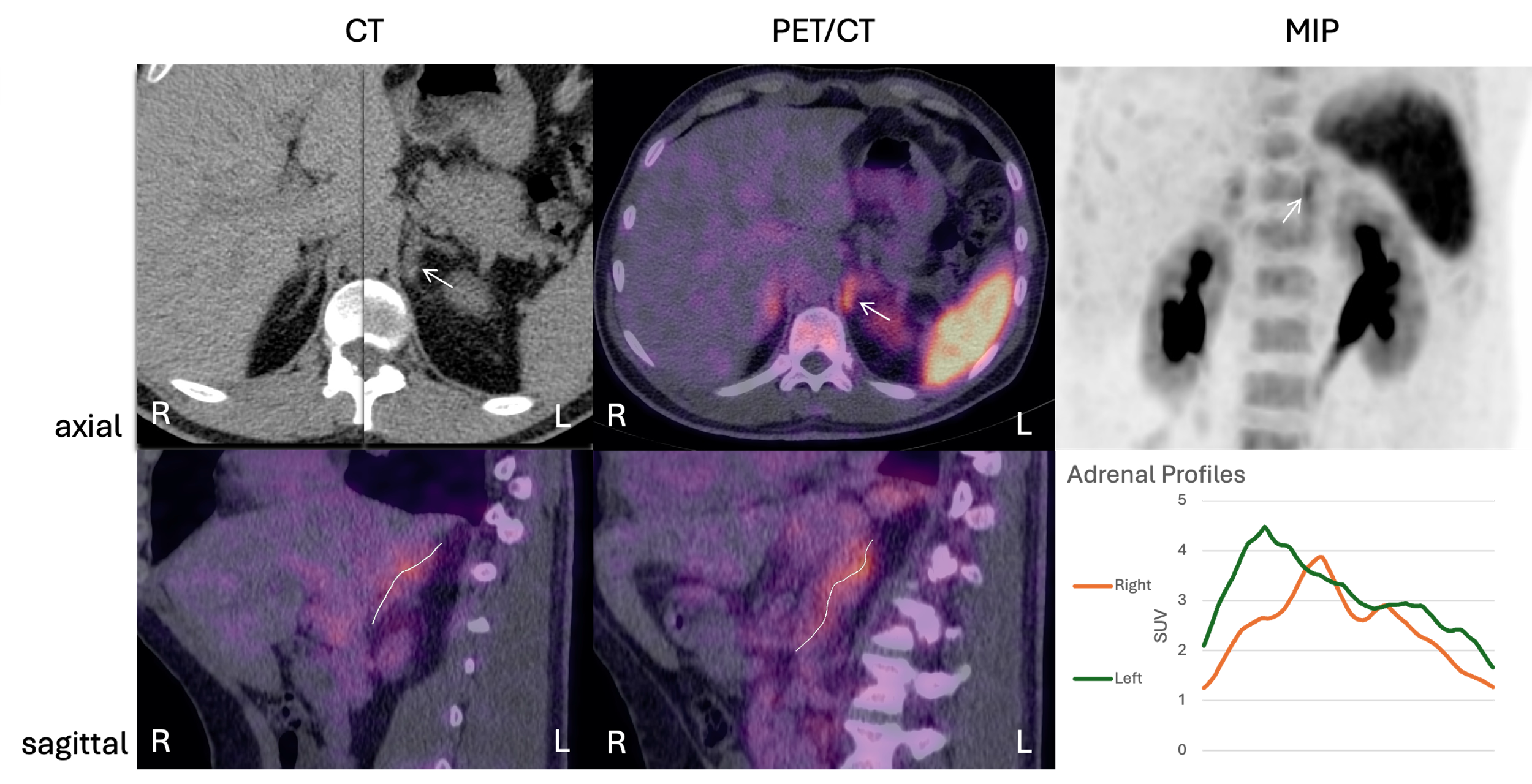


Multiplanar [^68^Ga]PentixaFor PET/CT images in patient 12 with partial biochemical success. The left adrenal gland shows no distinct morphological correlate on CT but mild tracer uptake (SUVmax ratio of 1.18 to the left). The local team initially reported the PET/CT as unilateral disease and the patient was referred to left adrenalectomy. In the retrospective re-evaluation, this patient received a PET/CT score of 2 after consensus from both centers. AVS was interpreted as ambiguous without reliable evidence of unilateral disease (Supplementary Table 1). Histopathology revealed a 9 mm aldosterone producing nodule and multiple smaller aldosterone producing nodules. Six months after surgery this patient showed partial biochemical and partial clinical success. White lines in fused sagittal PET/CT indicate the measurement path for the adrenal SUV profile. This case exemplifies [^68^Ga]PentixaFor PET/CT in bilateral, but asymmetric PA and potential inconsistencies in reporting [^68^Ga]PentixaFor PET/CT if standardized PET/CT criteria are lacking.

MIP, Maximum intensity projection

**Supplementary Figure 4.** Pathways of patients who had PET and AVS.


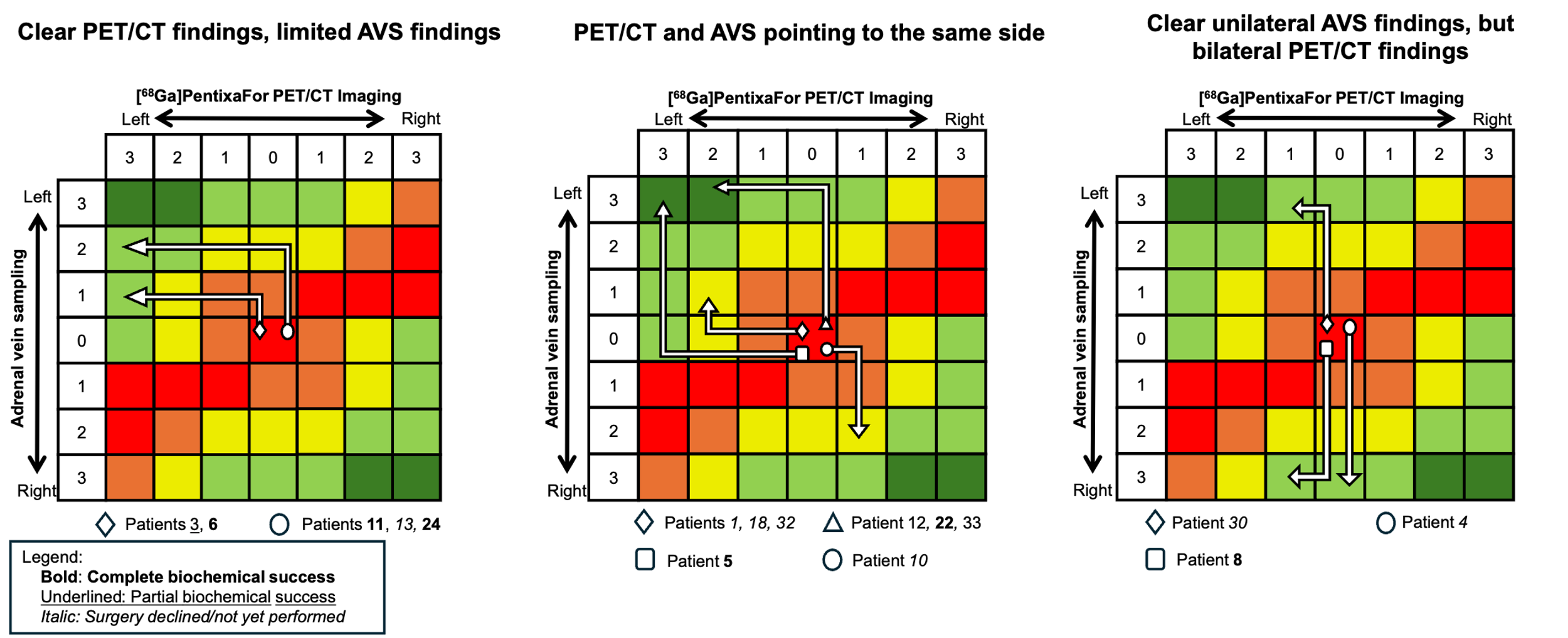


Pathways of 16/22 patients that underwent AVS and [^68^Ga]PentixaFor PET/CT are presented. From the remaining 6 patients, 3 patients were classified as bilateral or low probability for unilateral disease on both modalities. One more patient scored 0 on PET/CT and 2 on AVS, and in two patients PET/CT and AVS pointed to different sides (one patient scored 1 to the left on PET/CT and 2 to the right on AVS, and one patient scored 2 to the left on PET/CT and 3 to the right on AVS).

**Supplementary Table 1.** AVS of Patient 12.

| Localisation​ | Aldosterone in ng/l​ | Cortisol in nmol/l​ | Aldosterone/​Cortisol​ | |
| --- | --- | --- | --- | --- |
| 1) Left adrenal vein | 183.10 | 781 | | 0.23 |
| 2) Peripheral, parallel to 1)​ | 47.80 | 212 | | 0.23 |
| 3) Left adrenal vein | 309.00 | 829 | | 0.37 |
| 4) Peripheral, parallel to 3)​ | 56.70 | 196 | | 0.29 |
| 5) Right adrenal vein | 514.60 | 13195 | | 0.04 |
| 6) Peripheral, parallel to 5)​ | 64.00 | 341 | | 0.19 |
| 7) Right adrenal vein | 1018.00 | 17815 | | 0.06 |
| 8) Peripheral, parallel to 7)​ | 91.00 | 471 | | 0.19 |

AVS in patient 12 with partial biochemical remission. This non-ACTH stimulated AVS narrowly missed the criteria for apparent bilateral adrenal suppression and showed markedly different selectivity indices (left: 4.23, right: 37.82) in the absence of hypercortisolism. Therefore, the local team initially interpreted this AVS as ambiguous, providing no reliable evidence of unilateral disease but possible asymmetric disease left > right. The initial local PET/CT interpretation as unilateral disease therefore guided left adrenalectomy. However, a retrospective strict application of the proposed AVS criteria yielded an AVS score of 3 (LI of 6.17 and CSI of 0.32). The reappraisal with standardized PET/CT interpretation criteria assigned a PET score of 2 from both centers, which better reflects asymmetric bilateral PA consistent with the partial biochemical and clinical success after left adrenalectomy.

**Supplementary Table 2**. Literature review with the respective relevant parameters.

| Reference | Country, City | Design | Unilaterals (defined by) | Bilaterals (defined by) | AVS Concordance | Diagnostic performance | | | | | | | | Further semiquantification | Uptake Time (min) | Acquisition Time (min) |
| --- | --- | --- | --- | --- | --- | --- | --- | --- | --- | --- | --- | --- | --- | --- | --- | --- |
|  |  |  |  |  |  | Visual | | SUVmax-ratio | | | Dominant SUVmax | | |  |  |  |
|  |  |  |  |  |  | Sensitivity | Specificity | Best cut-off | Sensitivity | Specificity | Best cut-off | Sensitivity | Specificity |  |  |  |
| 1. Heinze et al., 2018 (1) | Germany, Würzburg & München | retrospective, multicenter | 9 (AVS) | 0 | 57% (4/7) |  |  |  |  |  |  |  |  |  | 50-69 | 20 |
| 2. Ding et al., 2020 (2) | China, Beijing | retrospective, single-center | 25 (pathology APA, 21 with complete biochem. success) | 11 ( 4 pathology, 7 "NFA") |  | 100% | 79% |  |  |  | 11.18 | 88% | 100% | LLR, LCR | 25-30 | 10 |
| 3. Ding et al., 2022 (3) | China, Beijing | retrospective, single-center | 43 (pathology) | 16 (pathology) | 50% (2/4) | 98% | 88% |  |  |  | 7.1 | 91% | 85% | LLR, LCR | 25-30 | 5 |
| 4. Chaman Baz et al., 2022 (4) | Netherlands, Nijmegen | prospective, single-center, registered | 15 (AVS) | 10 (AVS) | 68% (17/25) |  |  | 1.4 | 60% (for 25 AVS) | 80% (for 25 AVS) |  |  |  |  | 60 |  |
| 5. Gao et al., 2023 (5) | China, Beijing | prospective, single-center | 39 (pathology) | 21 (pathology) |  | 93% | 85% |  |  |  | 8.95 | 77% | 92% |  |  |  |
| 6. Hu et al., 2023 (6) | China, Chongqing | prospective, single-center, not-registered | 43 (AVS, pathology) | 57 (AVS) | 90% (90/100) |  |  | 1.65 (10min) 1.57 (40min) | 77% (10min) 86% (40min) | 100% (10min) 91% (40min) |  |  |  | DSAL | 10 & 40 | 5 |
| 7. Y. Zheng et al., 2023 (7) | China, Changsha | prospective, single-center | 66 (APA; pathology) | 33 (adrenal hyperplasia; orthostasis test, AVS, pathology) | 60% (3/5) | 92% | 94% |  |  |  | 7.65 | 85% | 90% | LLR, LAR | 30 | 8 |
| 8. Yin et al., 2024 (8) | China, Changsha | prospective, single-center | 19 (complete biochem. success) | 7 (AVS) | 77% (20/26) | 89% | 92% |  |  |  | 5.71 | 79% | 100% | LLR, LCR | 25 | 10 |
| 9. Ding et al., 2024 (9) | China, Beijing | prospective, single-center registered | 82 (68 PET only, 17 AVS and PET or follow up) | 22 (AVS) | 67% (24/36) | 90% | 86% | 1.35 | 71% (for 36 AVS) | 68% (for 36 AVS) | 4.55 | 85% | 86% | LLR, LAR | 30-45 | 10 |
| 10. Zuo, Liu, Li, et al., 2024 (10) | China, Chongqing | retrospective, single-center | 16 (pathology and complete biochem success) | 9 (AVS) | 65% (15/23) |  |  |  |  |  |  |  |  |  | 10 & 40 |  |
| 11. Zhang et al., 2024 (11) | China, Chongqing | retrospective, single-center | 128 (34 by AVS, 94 by complete biochem success) | 80 (AVS) | 78% (163/208) | 73% | 88% | 1.5 1.65 | 68% (1.5) 61% (1.65) | 91% (1.5) 96% (1.65) | 11 | 43% | 95% | DSAL | 10 |  |
| 12. Zuo, Liu, Ren, et al., 2024 (12) | China, Chongqing | retrospective, single-center | 42 (AVS) | 19 (AVS) |  | 74% | 53% |  |  |  | 8.17 (10min) 4.71 (40min) | 64% (10min) 86% (40min) | 90% (10min) 74% (40min) | MTV, TLCRE | 10 & 40 | 5 |
| 13. Zuo et al., 2025 (13) | China, Chongqing | retrospective, single-center | 91 (pathology) | 70 (AVS, pathology) | 71% (97/136) | 79% (10min) 75% (40min) | 70% (10min) 79% (40min) | 1.46 (10min) 1.59 (40min) | 76% (10min) 79% (40min) | 64% (both) | 6.75 (10min) 4.85 (40min) | 75% (10min) 85% (40min) | 94% (10min) 87% (40min) | LLR, LAR | 10 & 40 | 5 |
| 14. Yi et al., 2025 (14) | China, Beijing | prospective, single-center, not-registered | 28 (AVS) | 9 (AVS) | 86% (32/37) | 89% | 78% | 2.4 | 61% | 89% | 6.86 | 79% | 67% |  | 25-30 | 10 |
| 15. Lu et al., 2025 (15) | China, Wuhan | prospective, single-center, registered | 38 *(pathology)* | 12 (5 on AVS, 7 IHA in pathology) | 76% (28/37) | 74% | 100% |  |  |  | 5.85 | 82% | 73% |  | 30 | 10 |
| 16. G. Zheng et al., 2025 (16) | China, Beijing | prospective, single-center, registered | 91 *(76 APA, 15 MAPN in pathology, 51/85 complete biochem. success)* | 0 *(34/88 partial biochem. success, 3/88 biochem. failure, but 64/91 with partial adrenalectomy)* | 57% (8/14) | *Positive Detection Rate APA: 68/76 (90%) MAPN: 10/15 (67%)* |  |  |  |  |  |  |  |  | 20-40 |  |
| 17. W. Zheng et al., 2025 (17) | China, Fuzhou | prospective, single-center, registered | 50 *(with complete biochem. success)* | 40 *(with partial/absent biochem. success)* |  |  |  |  |  |  | 9.8 |  |  |  | 40-60 | 10 |
| 18. Lin et al., 2025 (18) | China, Nanjing | Retrospective, single-center | 47 APA *(pathology)* | 26 non functional adenoma *(clinical and PET)* |  | 100% | 100% |  |  |  | 8.98 | 100% | 95.7% | LLR, LCR | up to 70min |  |
| 19. Shao et al., 2025 (19) | China, Changsha | Prospective, single-center, registered | 60 *(pathology, and 57 with complete biochemical success)* | 41 *(PET only)* | 66% (14/21) |  |  |  |  |  |  |  |  | LLR, LCR *(SUVmean of contralateral gland)* | 25 | 10 |
| 20. Tan et al., 2025 (20) | China, Sichuan | Retrospective, single-center | 23 *(PET criteria, 21/23 with complete biochemical success)* | 0 |  |  |  |  |  |  | 6.5 *(pre-specified)* |  |  | LLR, LCR *(SUVmean of contralateral gland)* | 40-60 |  |
| 21. Gao et al., 2025 (21) | China, Beijing | Retrospective, single-center | 63 APA *(pathology)* | 16 IHA *(AVS, pathology and CT)* |  | 94% | 56% |  |  |  | 8.0 | 70% | 94% |  |  | 10 |
| 22. G. Zheng et al., 2025 (22) | China, Beijing | Prospective, single-center, registered | 110 (complete biochemical success) | 24 (partial or absent biochemical success) |  |  |  |  |  |  | 5.5 | 83% | 93% |  | 20-40 |  |
| 23. Shu et al., 2025 (23) | China, Changsha | Retrospective, single-center, registered | 32  (complete biochemical success) | 4  (incomplete biochemical success) | 80% (4/5) | 88% | 100% | 2.03 | 75% | 100% | 8.3 | 91% | 100% | LLR |  |  |
|  |  |  |  |  |  |  |  |  |  |  |  |  |  |  |  |  |

Abbreviations: AVS, adrenal vein sampling; MAPN, multiple aldosterone producing nodules; NFA, non-functioning adenoma; APA, aldosterone producing adenoma; LLR, lesion to liver ratio (SUVmean); LCR, lesion to contralateral adrenal ratio (SUVmean or SUVmax, not stated in publications); LAR, lesion to normal adrenal ratio (SUVmean); DSAL, dominant side adjusted by liver (SUVmean); MTV, metabolic tumor volume; TLCRE, total lesion CXCR4 expression

**Supplementary Figure 5**. PRISMA flow chart of included literature (24).


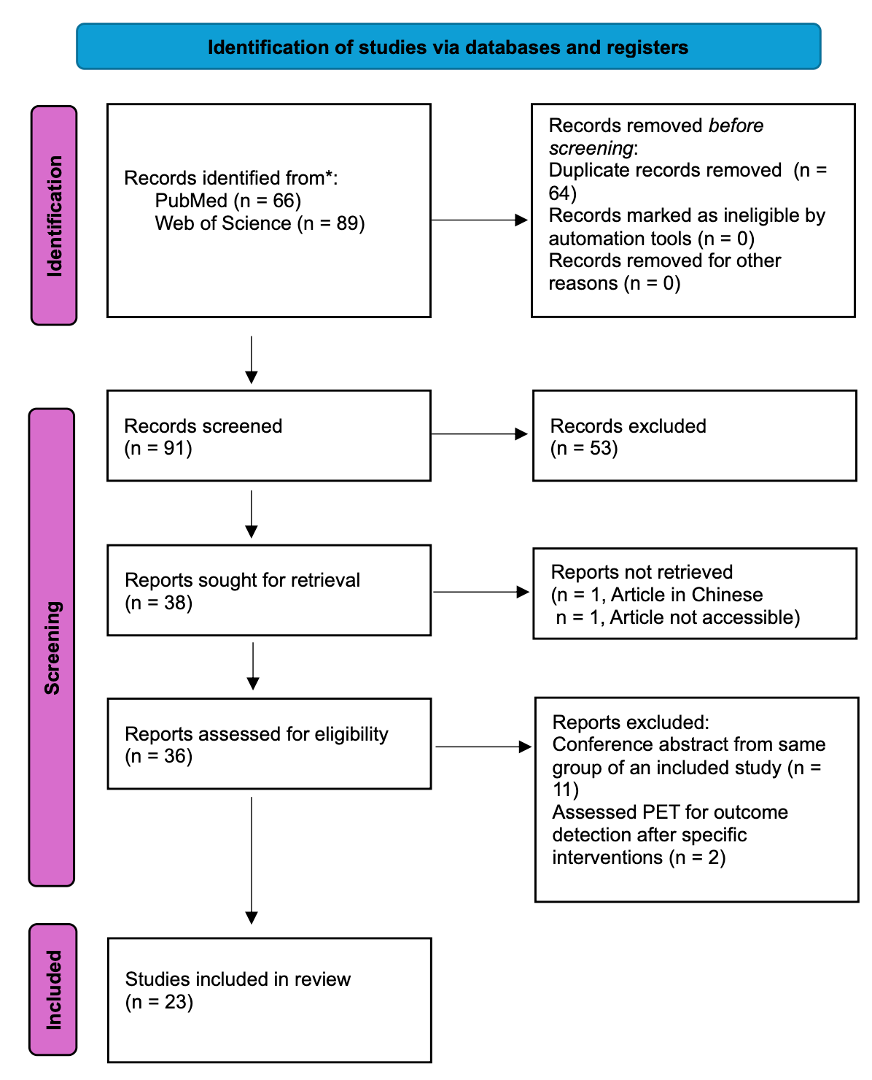


**References**

1. Heinze B, Fuss CT, Mulatero P, Beuschlein F, Reincke M, Mustafa M, et al. Targeting CXCR4 (CXC Chemokine Receptor Type 4) for Molecular Imaging of Aldosterone-Producing Adenoma. Hypertension. 2018 Feb;71(2):317–25.

2. Ding J, Zhang Y, Wen J, Zhang H, Wang H, Luo Y, et al. Imaging CXCR4 expression in patients with suspected primary hyperaldosteronism. Eur J Nucl Med Mol Imaging. 2020 Oct;47(11):2656–65.

3. Ding J, Tong A, Zhang Y, Wen J, Zhang H, Hacker M, et al. Functional Characterization of Adrenocortical Masses in Nononcologic Patients Using 68Ga-Pentixafor. J Nucl Med. 2022 Mar;63(3):368–75.

4. Baz AH in C, Gotthardt M, Spiering W, Deinum J, Langenhuijsen J. CXCR4-directed [68Ga]Ga-PentixaFor PET/CT as a diagnostic modality in subtyping primary aldosteronism. In: Endocrine Abstracts. European Congress of Endocrinology 2024, Stockholm, Sweden: Bioscientifica; 2024. p. P227.

5. Gao Y, Ding J, Cui Y, Li T, Sun H, Zhao D, et al. Functional nodules in primary aldosteronism: identification of CXCR4 expression with 68Ga-pentixafor PET/CT. Eur Radiol. 2023 Feb 1;33(2):996–1003.

6. Hu J, Xu T, Shen H, Song Y, Yang J, Zhang A, et al. Accuracy of Gallium-68 Pentixafor Positron Emission Tomography-Computed Tomography for Subtyping Diagnosis of Primary Aldosteronism. JAMA Netw Open. 2023 Feb 1;6(2):e2255609.

7. Zheng Y, Long T, Peng N, Zhen M, Ye Q, Zhang Z, et al. The Value of Targeting CXCR4 With 68Ga-Pentixafor PET/CT for Subtyping Primary Aldosteronism. J Clin Endocrinol Metab. 2023 Dec 21;109(1):171–82.

8. Yin X, Ai K, Luo J, Liu W, Ma X, Zhou L, et al. A comparison of the performance of 68Ga-Pentixafor PET/CT versus adrenal vein sampling for subtype diagnosis in primary aldosteronism. Front Endocrinol (Lausanne). 2024;15:1291775.

9. Ding J, Li X, Liu S, Gao Y, Zheng G, Hacker M, et al. Clinical Value of 68Ga-Pentixafor PET/CT in Subtype Diagnosis of Primary Aldosteronism Patients with Adrenal Micronodules. J Nucl Med. 2024 Jan 2;65(1):117–24.

10. Zuo R, Liu S, Li W, Xia Z, Xu L, Pang H. Clinical value of 68Ga-pentixafor PET/CT in patients with primary aldosteronism and bilateral lesions: preliminary results of a single-centre study. EJNMMI Res. 2024 July 4;14(1):61.

11. Zhang X, Song Y, Jing Y, Hu J, Shen H, Zhang A, et al. Comparison of Different Diagnostic Criteria of 68Ga-Pentixafor PET/CT for the Classification of Primary Aldosteronism. J Clin Endocrinol Metab. 2024 Nov 29;dgae747.

12. Zuo R, Liu S, Ren X, Li W, Xia Z, Xu L, et al. Typing diagnostic value of 68Ga-pentixafor PET/CT for patients with primary aldosteronism and unilateral nodules. Endocrine. 2024 Sept 9;

13. Zuo R, Liu S, Ren X, Li W, Xia Z, Xu L, et al. Clinical Utility of Dual-Time 68Ga-Pentixafor PET/CT in Diagnosing and Subtyping Primary Aldosteronism. Clinical Endocrinology. 2025;102(5):499–509.

14. Yi T, Lu D, Cui Y, Zhang Z, Yang X, Zhang J, et al. 68Ga-pentixafor PET/CT Is a Supplementary Method for Primary Aldosteronism Subtyping Compared with Adrenal Vein Sampling. Mol Imaging Biol. 2025 Feb;27(1):142–50.

15. Lu N, Chen L, Yu F, Xiao Z, Xing D, Zhong J, et al. Evaluating the value of chemokine receptor type 4–targeted PET imaging in diagnosing primary aldosteronism lateralization: A comparison with adrenal venous sampling. Surgery. 2025 May 1;181:109156.

16. Zheng G, Ding J, Gao Y, Liu S, Yan X, Wang W, et al. 68Ga-pentixafor PET/CT in guiding surgical management of primary aldosteronism. J Clin Transl Endocrinol. 2025 Mar;39:100384.

17. Zheng WC, Chen SM, Qiu QR, Li XD, Lin F, Shen XM, et al. Total or partial adrenalectomy for aldosterone-producing adenoma: can 68Ga-Pentixafor PET/CT predict surgical outcomes? Eur J Nucl Med Mol Imaging. 2025 Aug 1;52(10):3632–42.

18. Lin T, Song J, Wang F, Guo H. 68Ga-pentixafor PET/CT predict the surgical outcome for the primary aldosteronism. Clin Transl Imaging. 2025 June 1;13(3):223–30.

19. Shao S, Xu H, Xing Z, Hong Y, Yin X, Luo J, et al. The prognostic value of CXCR4 PET/CT imaging in unilateral primary aldosteronism patients after adrenalectomy. EJNMMI Research. 2025 Apr 17;15(1):41.

20. Tan L, Chen T, Zhang W, Shen S, Tian H, Zhu Y, et al. CXCR4-directed PET with 68Ga-pentixafor versus adrenal vein sampling for the diagnosis of unilateral primary aldosteronism. Endocrine. 2025 Aug 1;89(2):603–13.

21. Gao Y, Ding J, Wang Y, Zhou Y, Zhang Y, Qiu L, et al. Primary Aldosteronism Classification With 18-Oxocortisol and Gallium-68–Pentixafor Positron Emission Tomography/Computed Tomography. Endocrine Practice. 2025 May 1;31(5):614–9.

22. Tang BNT, Levivier M, Heureux M, Wikler D, Massager N, Devriendt D, et al. 11C-methionine PET for the diagnosis and management of recurrent pituitary adenomas. Eur J Nucl Med Mol Imaging. 2006 Feb 1;33(2):169–78.

23. Shu Z, He Y, Long T, Guo M, Xia Z, Fu X, et al. Is CXCR4-targeted 68Ga-pentixafor PET/CT a reliable AVS-free modality for surgical decision-making and prognostic prediction in primary aldosteronism with bilateral adrenal lesions? EJNMMI Res. 2025 Aug 28;15(1):111.

24. Page MJ, McKenzie JE, Bossuyt PM, Boutron I, Hoffmann TC, Mulrow CD, et al. The PRISMA 2020 statement: an updated guideline for reporting systematic reviews. BMJ. 2021 Mar 29;372:n71.
